# Tracking of Blood Pressure in Children and Adolescents in Germany in the Context of Risk Factors for Hypertension

**DOI:** 10.1155/2018/8429891

**Published:** 2018-09-26

**Authors:** Giselle Sarganas, Angelika Schaffrath Rosario, Claudia Niessner, Alexander Woll, Hannelore K. Neuhauser

**Affiliations:** ^1^Robert Koch Institute, Department of Epidemiology and Health Monitoring, Berlin, Germany; ^2^DZHK (German Center for Cardiovascular Research), Partner Site Berlin, Germany; ^3^Institute for Sports and Sport Science, Karlsruhe Institute of Technology, Karlsruhe, Germany

## Abstract

Blood pressure (BP) tracking from childhood to adulthood has two aspects: the ranking stability relative to others over time and the prediction of future values. This study investigates BP tracking in children and adolescents in Germany in the context of hypertension risk factors. BP was measured and analyzed in 2542 participants of the German Health Examination Survey for Children and Adolescents (t_0_ 2003-2006; 3 to 17-year olds) and of a six year follow-up “Motorik Modul” (t_1_ 2009-2012; 9 to 24-year olds). BP tracking coefficients were calculated from Spearman's rank-order correlations. Predictive values and logistic regression models were used to forecast t_1_-BP above the hypertension threshold from t_0_-BP as well as from baseline and follow-up hypertension risk factors. BP tracking was moderate (0.33-0.50 for SBP and 0.19-0.39 for DBP) with no statistically significant differences between sex and age groups. Baseline hypertensive BP was the strongest independent predictor of hypertensive BP at follow-up (OR 4.3 and 3.4 for age groups 3-10 and 11-17 years) after adjusting for sex, BMI trajectories, birthweight, parental hypertension, and age-group dependent-sports/physical activity. However, the positive predictive value of baseline hypertensive BP for hypertensive BP at follow-up in 3- to 10-year olds was only 39% (34% in 11- to 17-year olds) and increased only moderately in the presence of additional risk factors. Our analysis with population-based data from Germany shows that BP in children and adolescents tracks only moderately over six years. BP in childhood is the strongest independent predictor of future BP but its predictive value is limited.

## 1. Introduction

Blood pressure (BP) elevation is much more common among children and adolescents than previously thought. Moreover, BP in childhood correlates with BP in adulthood [1]; hence children with elevated BP have a higher probability of developing hypertension in adulthood than children with lower BP. However, the usefulness of universal pediatric BP screening is strongly debated due to the only moderate BP tracking correlation from childhood to adulthood [1–4].

BP tracking correlation has two aspects: the stability of BP ranking relative to others over a specified period of time (calculated as tracking coefficients) and the predictability of future values from baseline values. Meta-analyses have reported moderate tracking coefficients of 0.42-0.38 for SBP and 0.41-0.28 for DBP from childhood to adulthood [1, 3]. While several studies have investigated BP tracking in children and adolescents and have compared different age groups as well as BP tracking over different time periods, little is known on whether having risk factors for hypertension, such as overweight and obesity, low birthweight, or parental hypertension, is associated with higher BP tracking correlation from childhood to adolescence/young adulthood. Moreover, the predictability of BP measurements from hypertension risk factors in clinically meaningful terms such as positive and negative predictive values has been reported only in the context of screening, not of tracking [5].

Therefore, the aim of this study is twofold: (1) to investigate six year BP tracking correlation coefficients from childhood to adolescence/young adulthood in a large population-based sample with a broad baseline age range of 3 to 17 years and to illustrate whether this aspect of tracking is stronger in subgroups with additional hypertension risk factors; and (2) to analyse the predictability of future BP values above the hypertension threshold from previous BP and hypertension risk factors and to quantify this predictability using different measures of association including odds ratios (OR) and predictive values.

## 2. Methods

### 2.1. Study Population

The German Health Interview and Examination Survey for Children and Adolescents (KiGGS) is a nationwide study based on a stratified population-registry sample of 17,641 participants aged 0–17 years living in Germany (response rate 67%), including 14,835 participants aged 3-17 years [6]. Details on the baseline and follow-up surveys including its several parallel study modules were published previously [6–8]. At baseline, about half of the participants were invited to take part in the “Motorik Modul Study” (MoMo; response rate 58%) [7]. The six-year MoMo follow-up had a response rate of 48% [7]. The present analysis includes data from 2686 children and adolescents who participated in the KiGGS baseline study KiGGS0 (t_0_ 2003-2006; participants aged 3 to 17 years) and the MoMo follow-up (t_1_ 2009-2012; participants aged 9 to 24 years) with complete BP data sets and who have not taken antihypertensive medication at t_0_ (unfortunately we do not have information on medication use at t_1_). The proportion of complete data sets was higher for those 3-10 years old at t_0_ than for those 11-17 years old. Analyses were stratified by age group: 3-10 years at t_0_ and 9-17 years at t_1_ vs. 11-17 years at t_0_ and 18-24 years at t_1_. We therefore excluded 144 participants who did not fall into these two groups in order to have a clear cutoff in the age group analysis and not mix both hypertension definitions (percentile and mmHg based; see below), leaving 2542 participants (1269 males and 1273 females) for analysis. Additional follow-up data were available from the telephone interview KiGGS1 2009-2012 [8].

BP measurement methods were standardized and followed the same protocol in KiGGS0 2003-2006 (t_0_) and in the MoMo study 2009-2012 (t_1_). At both time-points, BP measurements were taken in the sitting position on a height-adjustable chair with a backrest, the right forearm resting on a table at the level of the heart, the elbow slightly bent, the legs uncrossed, and the feet placed firmly on the floor. Four cuff sizes (cuff bladder in cm 6 × 12, 9 × 18, 12 × 23, or 17 × 38.6) were available, which had to cover at least two-thirds of the upper arm length (from the axilla to the antecubital fossa). Two readings of systolic (SBP), diastolic blood pressure (DBP), mean arterial BP, and heart rate were obtained at a two-minute interval with an automated upper arm oscillometric device (Datascope Accutorr Plus, Mahwah, NJ) after a nonstrenuous part of the examination and an additional 5-minute rest in both surveys [9, 10]. The mean of the 2 systolic and diastolic measurements was used for this analysis.

BP in children is age, gender, and height dependent; therefore, it is not possible, as for adults, to designate a single BP threshold to define hypertension. BP reference percentiles [11] based on KiGGS data from nonoverweight children were used according to German guidelines [12]: hypertensive BP measurement was SBP or DBP at or above the 95th age-, sex-, and height-specific percentile for children and adolescents under 18 years, or BP ≥ 140/90 mmHg irrespective of the percentile. For young adults aged 18 years or older, the absolute cutoff for hypertension (≥140/90 mmHg) was applied. Since percentile-based cutoffs in children and the 140/90 mmHg cutoff in adults are not equivalent, analyses were stratified accordingly: baseline ages 3 to 10 years with t_1_-ages 9 to 17 years vs. baseline ages 11 to 17 years with t_1_-ages 18 to 24 years.

In addition, weight and height were measured at t_0_ and t_1_ according to a common protocol. Weight was measured in underwear to the nearest 0.1 kg with a calibrated scale (Seca, Birmingham, United Kingdom) according to a standardized protocol [6]. BMI was calculated as the ratio of weight (in kg) by height^2^ (in m^2^) and rounded to 3 digits. BMI was categorized as underweight, normal weight, overweight, and obese according to the International Obesity Task Force (IOTF) cutoffs defined by the age-specific percentiles in <18-year olds, which at the age 18 and older correspond to the adult cutoff points for overweight 25 kg/m^2^ and obesity 30 kg/m^2^ [13]. BMI trajectory was defined as the BMI course between t_0_ and t_1_ in 4 categories: “persistently low or normal” defined as under- or normal weight at t_0_ and at t_1_; “resolution” as overweight or obese at t_0_ and normal or underweight at t_1_; “persistently overweight/obesity” (at t_0_ and at t_1_); and “incident overweight/obesity” as under- or normal weight at t_0_ and overweight or obese at t_1_.

Information on physical activity was collected only in t_0_: in children aged 3 to 10 years it was assessed through parent-reported frequency of sports activities per week (categories: 3 or more, 1-2, and less than once) [14] and in children aged 11 to 17 years through self-reported leisure time physical activities that makes them sweat or get out of breath (categories: ≤2.0, 2.1-4.9, and 5.0-31.0 hours per week) [15].

Birth weight (in g) was reported by the mother at t_0_; we have classified the variable into less than 2500 g, as low birth weight, or greater than 2500 g.

Parental hypertension was collected at t_1_ with the question: has the mother or father of the study participant ever been diagnosed by a doctor as having hypertension or high blood pressure?

### 2.2. Statistical Analysis

The analysis was performed in two parts. First, BP tracking was estimated by calculating Spearman's rank-order correlation coefficients stratified by sex and age and potential hypertension risk factors (overweight/obesity, birthweight and parental hypertension). For this purpose, four age groups were chosen: 3 to 6, 7 to 10, 11 to 14, and 15 to 17 years. In the first two age groups, the rank order of t_0_-percentiles was correlated with the rank order of t_1_-percentiles. For the last two groups, the rank order of t_0_-percentiles were correlated with the rank order of t_1_ mmHg values.

For testing statistical differences between correlation coefficients, we have used the software cocor (http://comparingcorrelations.org/) [16].

In the second part of the analysis, we determined the proportion of participants with hypertensive BP measurement at t_0_ and t_1_. For calculating the positive predictive value (PPV) of hypertensive BP at t_0_ for future hypertensive BP (six years later at t_1_), as well as the negative predictive value (NPV) of normotensive BP at t_0_ for future normotensive BP, we used the software MedCalc (https://www.medcalc.org/calc/diagnostic_test.php). We calculated the PPV and NPV also for risk factor combinations, e.g., for having hypertensive BP and being obese at t_0_. In sensitivity analyses, we checked whether the results were altered when using weighting factors in the analysis. These weights take selective participation in the MoMo study and the drop-out between t_0_ and t_1_ into account. They are the inverse of the probability to participate in the MoMo study/the follow-up, estimated in a logistic regression model including several relevant KiGGS variables as predictors. Since no weights are available for the exact population under study here, we used three existing sets of weights: longitudinal weights adjusting the KiGGS follow-up to the KiGGS baseline, longitudinal weights adjusting the MoMo follow-up to the MoMo baseline, and cross-sectional MoMo weights adjusting the MoMo follow-up to the KiGGS1 follow-up.

Furthermore, we investigated the prediction of hypertensive t_1_-BP from t_0_-BP and other baseline and follow-up risk factors for hypertension, using logistic regression models. Models were run separately for the t_0_-age-group 3 to 10 years (percentile-defined t_1_-hypertension) and the t_0_-age-group 11 to 17 years (140/90 mmHg threshold t_1_-hypertension). STATA SE14.1 was used for the analyses.

## 3. Results

The characteristics of the 2542 study participants (1269 males and 1273 females) at baseline (2003-2006) and six year follow-up (2009-2012) are presented in Table 1. At six-year follow-up, mean SBP had significantly increased by 14.8 mmHg in males and by 11.9 mmHg in females. In addition, the proportion of overweight in males and obesity in both sexes was at follow-up statistically significantly greater compared to baseline (Table 1).

Spearman rank-order correlations for BP tracking from childhood to adolescence/young adulthood are shown in Table 2. Tracking coefficients were higher for SBP (ranging for sex-age groups from 0.33 to 0.50) than for DBP (ranging for sex-age groups from 0.19 to 0.39). Although tracking coefficients increased with age in males, this increase was not statistically significant. Other differences between sex and age groups were as well not statistically significant.

The BP tracking coefficients stratified by birthweight, parental hypertension and BMI trajectories remained in the order of magnitude of the tracking coefficients for the overall sex-age groups; i.e., children with these hypertension risk factors did not have substantially higher SBP or DBP tracking correlation coefficients compared to children without these risk factors (data not shown).

**Figure 1:**
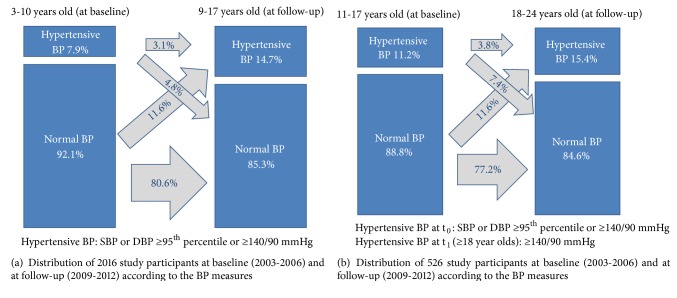
Blood pressure in 2542 study participants at baseline (2003-2006) and at follow-up (2009-2012).

We investigated the prevalence of hypertensive BP measurement at baseline and at follow-up in a younger age group (baseline age 3 to 10 years, pediatric percentile-based definition of hypertensive BP at t_1_) and an older age group (baseline age 11 to 17 years, adult definition of hypertensive BP at t_1_) (Figure 1). Among children aged 3 to 10 years, 7.9% (95% CI 6.7-9.1) had hypertensive BP at baseline. Of these, 39% (n = 62 out of 159) also had hypertensive BP at follow-up. The proportion of children with persistent hypertensive BP (at t_0_ and t_1_) accounted for 3.1% of this age group; incident hypertensive BP (children with normal BP at t_0_ and hypertensive BP measurement at t_1_) was observed in 11.6%; resolution of hypertensive BP (children with hypertensive BP at t_0_ and a normal BP at t_1_) was observed in 4.8% and the proportion of persistently normal BP in the 3- to 10-year-old group accounted for 80.5%. Among children and adolescents aged 11 to 17 years, 11.2% (95% CI 8.6-14) had hypertensive BP at baseline. Of these, 34% (n = 20 out of 59) also had hypertensive BP at follow-up. In this age group, persistent hypertensive BP accounted for 3.8%; incident hypertensive BP was observed in 12%; resolution of hypertensive BP in 7.4% and persistently normal BP in 77% of the study population (Figure 1).

The univariate and multivariable logistic regression (Table 3) showed the factors associated with having hypertensive BP at t_1_. In the models for both the younger and the older age group hypertensive BP at t_0_ was one of the major determinants (OR 4.25 (95% CI 2.90-6.23) and OR 3.35 (95% CI 1.60-7.01)). Further, increasing age was also identified as a risk factor for hypertensive BP at follow-up in both age groups. In the 3- to 10-year-old group, children with a persistently overweight/obesity as well as those with an incident overweight/obesity were more likely to have hypertensive BP at t_1_ (OR 2.70 (95% CI 1.78-4.12) and OR 4.44 (95% CI 3.08-6.42), respectively) compared to children who had a persistently low or normal BMI. A gender difference was found only in the 11- to 17-year-old group, in which females were less likely to have hypertensive BP at t_1_ compared with their male peers. Moreover, only in the 11- to 17-year-old group, parental hypertension was found to be a determinant for hypertensive BP at follow-up.

**Figure 2:**
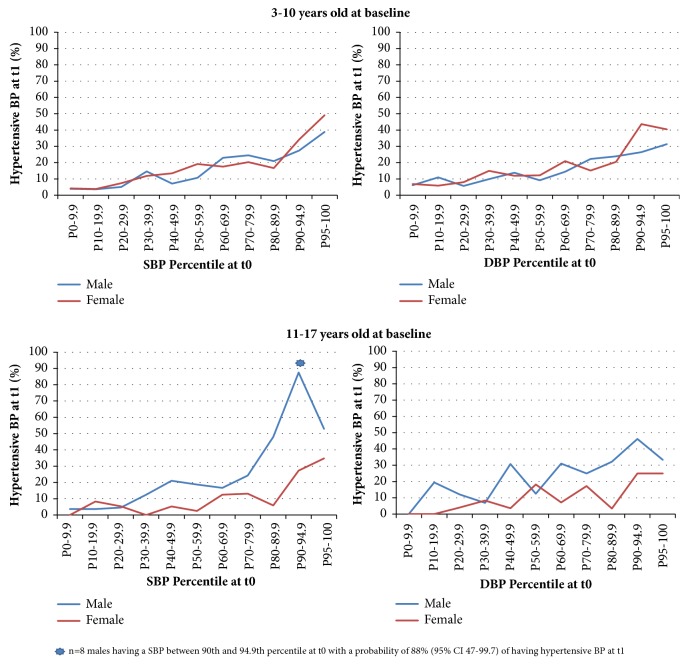
Probability of having hypertensive BP at six year follow-up (t1) according to baseline BP percentiles.

The probability of having hypertensive BP at six year follow-up increased with baseline BP percentile (Figure 2) but did not exceed 50% with the exception of a small subgroup (n = 8) of 11- to 17-year-old males having a SBP between 90th and 94.9th percentile (prehypertensive at t_0_) with a probability of 88% (95% CI 47-99.7) of having hypertensive BP at t_1_.

The positive (PPV) and negative predictive values (NPV) according to age at baseline and risk factors are presented in Table 4. The PPV of baseline hypertensive BP for hypertensive BP six years later was 39% (95% CI 32-46) for 3- to 10-year-old children and 34% (95% CI 24-45) for 11- to 17-year olds. The NPV of non-hypertensive baseline BP was 87% (95% CI 87-88) and 87% (95% CI 85-88) respectively. In the 3- to 10-year-old group, the PPV increased as risk factors were combined: for instance, 53% (95% CI 30-76) of 3- to 10-year-old children with a hypertensive BP and obesity at baseline also had hypertensive BP six years later. In the 11- to 17-year-old group, similar probabilities of having hypertensive BP at follow-up were observed in obese children at baseline (50%; 95% CI 27-74) and children with a hypertensive BP at baseline and parental hypertension (48%; 95% CI 30-67) (Table 4).

## 4. Discussion

This study investigates different aspects of BP tracking from childhood to adolescence/young adulthood with recent population-based data from Germany. Applying various metrics for assessing tracking including correlation coefficients, positive predictive values, and ORs from multivariable predictive modelling, BP tracking from childhood to adolescence and young adulthood was generally moderate. Our results confirm previous findings that BP in childhood and adolescence is one of the strongest predictors of future BP; however, it is one of the strongest predictors among several moderate or weak predictors. Among children and adolescents aged 3 to 17 years with hypertensive BP at baseline (mean of two measurements taken on one occasion), about one in three had hypertensive BP six years later. In population subgroups of children with additional hypertension risk factors such as overweight or parental hypertension, the PPV of baseline hypertensive BP increased but was not much higher than 50%.

In line with previous findings, we found moderate BP tracking coefficients, higher for SBP than for DBP [1–3]. Stratified by age and sex, we found a SBP tracking correlation in the range of 0.33 to 0.50 and for DBP in the range of 0.19 to 0.39, which was similar to the meta-analysis of Chen et al. [1] who found a mean correlation coefficient of 0.38 for SBP and of 0.28 for DBP.

One could ask whether BP tracking coefficients become larger among children with additional risk factors for hypertension, but in fact mathematically they are expected to remain similar or even become smaller, as verified with data from this study (results not shown). This happens since in subgroups with additional hypertension risk factors, BP values are more similar than in an unselected group and ranking to a greater extent due to chance. In other words, for combinations of risk factors, tracking coefficients are not an appropriate measure for assessing the predictability of future BP values.

Our results confirm previous findings that risk factors for hypertensive BP in young adulthood can be identified in childhood and adolescence [2, 17]. However, the associations were moderate. After adjusting for various hypertension risk factors including overweight/obesity and parental hypertension, a child aged 3 to 10 years with hypertensive BP was more likely to have hypertensive BP six years later than a child in the same age group with a normal BP (OR 4.25, 95% CI 2.90-6.23). For the age group 11 to 17 years the respective adjusted OR was 3.35 (95% CI 1.60-7.01).

The association between overweight/obesity and hypertension in children was observed in several studies [18–20]. Our study showed that 36% of 3- to 10-year-old and 50% of 11- to 17 year-old obese children at baseline had a hypertensive BP at follow-up. The multivariable logistic regression analyses revealed that 3- to 10-year-old participants with a persistently or incidentally overweight/obesity were more likely to have a hypertensive BP at follow-up compared to participants with a persistently low or normal BMI trajectory, suggesting a potential action for prevention of future hypertension. In this regard, other studies have shown that children with higher BMI at follow-up changed BP standard deviation scores from initially normal to higher BP values [21]. Moreover, it has been observed that changes in BMI during growth predicted BP levels during adolescence [22]; for instance, the study of Kelly et al. [2] showed that the resolution of elevated BP in the transition from childhood to adulthood could be partially explained by improvements in associated factors such as by decreasing the BMI z-score between childhood and adulthood.

Our univariable logistic regression analysis showed that children with hypertensive parents were at higher risk of hypertension at follow-up compared to children without hypertensive parents. However, this was only confirmed in the multivariable analysis of 11-17-year olds. Children and adolescents with parental hypertension had an 18% and 21% probability of having a hypertensive BP at follow-up (Table 4). A familial predisposition for hypertension was reported in previous studies [23, 24]; furthermore, it has been evaluated whether BP screening should target only children with a parental hypertension instead of all children (universal screening), with the conclusion that this may not be a substantially better strategy [5].

In our data, obesity of the child/adolescent at t_0_ was a significantly stronger predictor for hypertension at t_1_ than parental hypertension in both age groups (Table 4).

Consistent with the literature [25–27] we observed male sex as a risk factor for hypertension in young adults: Figure 2 showed that 11- to 17-year-old males at baseline had a higher probability for having a hypertensive BP at follow-up (18 to 24 years) compared to females and Table 3 presented an OR = 0.33 (95% CI 0.18-0.61) for 11- to 17-year-old females at baseline, where they were less likely to have a hypertensive BP at follow-up compared to their male peers.

Contrary to other reports [28, 29], our study did not find a significant association between low birthweight and hypertension.

Even though we did not find significant associations between physical activity at t_0_ and hypertensive BP at follow-up, our estimates showed the tendency of a negative correlation between these two variables, which is in line with other studies [30, 31].

Our study does not intend to estimate the prevalence of hypertension in children, as the diagnosis of hypertension requires, among other things, BP measurements on several occasions; however, the proportion of children and adolescents with persistently elevated BP in our study was 3.1% for 3- to 10-year olds at baseline and 3.8% for 11- to 17-year olds, which is in line with studies reporting prevalences of sustained hypertension in children and adolescents of 1% to 5% [32–34].

Major strengths of this study are the large national population database, coverage of a wide age range (3 to 17 years), the six-year follow-up, and standardized BP measurements according to the same protocol at baseline and follow-up examinations. However, despite using the same BP measurement devices, identical resting times, and the same measurement protocol, including performing BP measurements before motor performance testing, we cannot exclude the possibility that the environment and expectation of an extensive motor testing like in the MoMo study could have influenced BP at follow-up, leading to somewhat higher BP values and somewhat lower tracking indices. Another limitation is the possible selection bias arising from selective participation in the MoMo study and from longitudinal drop-out. Sensitivity analyses with weights that partially account for this selection showed somewhat lower predictive values and thus did not alter the main conclusions. A further limitation is that the rule for KiGGS0 (2003-2006) was that the cuff should cover at least two-thirds of the upper arm length while current recommendations (2016 ESH guidelines [4]) suggest that the cuff size should cover 80-100% of the individual's arm circumference. Another limitation is that we had only two time points for assessment of the tracking; however, the KiGGS study just completed its second wave, allowing a future analysis of BP trajectories over a 10-year follow-up [35].

In conclusion, our analysis with recent population-based data from Germany shows that BP in children and adolescents tracks only moderately over six years; however, BP in childhood is the strongest independent predictor for future hypertensive BP measurements.

## Figures and Tables

**Table 1:** Characteristics of the 2542 study participants (1269 males and 1273 females) at baseline (2003-2006) and at follow-up (2009-2012).

	Males			Females		
Characteristics Age range (years)	Baseline (t_0_) (3-17)	Follow-up (t_1_) (9-24)	p-value	Baseline (t_0_) (3-17)	Follow-up (t_1_) (9-24)	p-value
Mean age^*∗*^	8.5 (8.3-8.7)	14.7 (14.5-14.9)	0.000	8.5 (8.3-8.7)	14.8 (14.6-15.0)	0.000
SBP (mmHg)^*∗*^	102.8 (102.2-103.4)	117.6 (116.8-118.5)	0.000	102.8 (102.3-103.4)	114.7 (114.0-115.4)	0.000
DBP (mmHg)^*∗*^	62.1 (61.7-62.5)	66.2 (65.7-66.7)	0.001	62.7 (62.3-63.1)	66.0 (65.5-66.5)	0.001
Hypertensive BP^&^	8.5% (7.1-10.2)	15.7% (13.8-17.8)	0.001	8.6% (7.2-10.3)	14.0% (12.2-16.0)	0.000
BMI (kg/m^2^)^*∗*^	17.1 (16.9-17.2)	20.7 (20.4-20.9)	0.000	17.1 (17.0-17.3)	20.5 (20.3-20.8)	0.000
Underweight	9.8% (8.3-11.6)	6.1% (4.9-7.5)	0.001	11.3% (9.7-13.2)	9.4% (7.9-11.1)	0.104
Normal-weight	78.9% (76.6-81.1)	72.5% (70.0-74.9)	0.000	74.0% (71.6-76.4)	72.6% (70.0-75.0)	0.372
Overweight	9.1% (7.6-10.8)	16.3% (14.4-18.5)	0.000	12.2% (10.5-14.1)	13.8% (12.0-15.8)	0.238
Obese	2.2% (1.5-3.2)	5.1% (4.0-6.5)	0.000	2.4% (1.7-3.4)	4.3% (3.3-5.5)	0.011
Sports activities (3 or more times/week) (3-10 years)	43.2% (40.1-46.4)	-	-	35.5% (32.4-38.6)	-	-
Sports activities (h/week)^*∗*^ (11-17 years)	7.6 (6.5-8.8)	-	-	5.2 (4.7-5.7)	-	-
Parental hypertension	-	28.1% (25.7-30.8)	-	-	30.3% (27.7-32.9)	-
Low birthweight (<2500 g)	5.0% (3.9-6.4)	-	-	6.3% (5.1-7.8)	-	-

^*∗*^mean (95% CI).

^&^SBP or DBP at or above the 95th age-, sex-, and height-specific percentile for children and adolescents under 18 years, or BP ≥140/90 mmHg irrespective of the percentile. For young adults aged 18 years or older, the absolute cutoffs for hypertension (≥140/90 mmHg) were applied.

**Table 2:** Spearman rank-order correlation coefficients for six year-tracking of BP in children and adolescents in Germany.

Baseline age	n	Tracking coefficients	
		SBP	DBP
3-6 years^1^			
All	1063	0.38	0.24
Male	530	0.33	0.19
Female	533	0.44	0.30
7-10 years^1^			
All	953	0.42	0.36
Male	481	0.41	0.30
Female	472	0.42	0.42
11-14 years^2^			
All	320	0.34	0.34
Male	155	0.43	0.32
Female	165	0.36	0.38
15-17 years^2^			
All	206	0.47	0.36
Male	103	0.50	0.34
Female	103	0.46	0.39

^1^t_0_-BP and t_1_-BP in age-, sex-, and height-specific percentiles; ^2^t_0_-BP in percentiles and t_1_-BP in mmHg.

All Spearman rank-ordercorrelation coefficients were statistically significant at a p-value <0.001.

**Table 3:** Predictors of hypertensive BP^*∗*^ at six-year follow-up in children and adolescents in Germany.

	**univariable analysis**		**multivariable analysis**	
**3- to 10-year-olds (at baseline)**	**OR (95% CI)**	**p-value**	**OR (95% CI)**	**p-value**
Sex				
Male	Ref	0.50	Ref	0.19
Female	1.09 (0.85-1.39)		1.21 (0.91-1.59)	

Age	1.14 (1.08-1.21)	<0.001	1.15 (1.08-1.23)	<0.001

BP t_0_				
normal (<P95)	Ref	<0.001	Ref	<0.001
hypertensive (≥P95)	4.44 (3.14-6.28)		4.25 (2.90-6.23)	

BMI trajectory (t_0_ to t_1_)				
persistently low or normal	Ref	<0.001	Ref	<0.001
resolution	1.74 (0.96-3.16)		1.64 (0.86-3.11)	
persistently overweight/obesity	3.87 (2.67-5.61)		2.70 (1.78-4.12)	
incident overweight/obesity	3.95 (2.83-5.51)		4.44 (3.08-6.42)	

Sport activities (times/week)				
<1	Ref	0.14	Ref	0.07
1-2	1.21 (0.85-1.73)		1.30 (0.87-1.92)	
≥3	1.42 (0.99-2.02)		1.58 (1.06-2.35)	

Parental hypertension				
No	Ref	0.02	Ref	0.48
Yes	1.38 (1.06-1.80)		1.11 (0.83-1.49)	

Low birthweight (<2500g)				
No	Ref	0.59	Ref	0.69
Yes	1.14 (0.70-1.88)		1.12 (0.64-1.96)	

**11- to 17-year-olds (at baseline)**				

Sex				
Male	Ref	<0.001	Ref	<0.001
Female	0.37 (0.22-0.61)		0.33 (0.18-0.61)	

Age	1.22 (1.07-1.40)	<0.01	1.21 (1.03-1.42)	0.02

BP t_0_				
normal (<P95)	Ref	<0.001	Ref	0.001
hypertensive (≥P95)	3.41 (1.87-6.23)		3.35 (1.60-7.01)	

BMI trajectory (t_0_ to t_1_)				
persistently low or normal	Ref	0.07	Ref	0.32
resolution	1.82 (0.58-5.70)		0.85 (0.17-4.19)	
persistently overweight/obesity	2.23 (1.19-4.16)		1.96 (0.89-4.29)	
incident overweight/obesity	1.50 (0.73-3.08)		1.59 (0.65-3.88)	

Physical activities (hours/week)				
≤2.0	Ref	0.24	Ref	0.42
2.1-4.9	1.76 (0.76-4.07)		1.75 (0.69-4.40)	
5.0-31.0	1.93 (0.90-4.13)		1.70 (0.74-3.91)	

Parental hypertension				
No	Ref	0.03	Ref	0.04
Yes	1.78 (1.07-2.95)		1.85 (1.02-3.33)	

Low birthweight (<2500g)				
No	Ref	0.46	Ref	0.56
Yes	0.57 (0.13-2.50)		0.62 (0.12-3.16)	

^*∗*^Hypertensive BP: SBP or DBP at or above the 95th age-, sex-, and height-specific percentile for children and adolescents under 18 years or BP ≥140/90 mmHg irrespective of the percentile; for adults aged 18 or older: ≥140/90 mmHg.

OR = odds ratio BP = blood pressure.

**Table 4:** Positive (PPV) and negative predictive value (NPV) of various hypertension risk factors or risk factor combinations for hypertensive BP^*∗*^ at six-year follow-up.

	n	Proportion at t_0_ % (95% CI)	PPV % (95% CI)	NPV % (95% CI)
**Baseline age 3 to 10 years**				

Hypertensive BP at t_0_	2016	7.9 (6.8-9.1)	39 (32-46)	87 (87-88)
Overweight or obesity at t_0_	2011	12 (11-14)	27 (22-32)	87 (86-88)
Obesity at t_0_	2011	2.2 (1.7-3.0)	36 (23-50)	86 (85-86)
Parental hypertension	1942	29 (27-31)	18 (15-20)	87 (86-88)
Hypertensive BP and overweight or obesity at t_0_	2011	2.0 (1.5-2.7)	48 (33-62)	86 (86-86)
Hypertensive BP and obesity at t_0_	2011	0.7 (0.4-1.2)	53 (30-76)	86 (85-86)
Hypertensive BP at t_0_ and parental hypertension	1942	2.6 (2.0-3.4)	51 (38-64)	86 (86-87)

**Baseline age 11 to 17 years**				

Hypertensive BP at t_0_	526	11 (8.8-14)	34 (24-45)	87 (85-88)
Overweight or obesity at t_0_	525	17 (14-20)	24 (17-33)	87 (85-88)
Obesity at t_0_	525	2.7 (1.6-4.5)	50 (27-74)	86 (85-87)
Parental hypertension	481	32 (28-36)	21 (16-26)	87 (85-89)
Hypertensive BP and overweight or obesity at t_0_	525	3.2 (2.0-5.1)	35 (17-59)	85 (85-86)
Hypertensive BP and obesity at t_0_	525	0.6 (0.2-1.8)	§	85 (85-85)
Hypertensive BP at t_0_ and parental hypertension	481	4.8 (3.2-7.1)	48 (30-67)	86 (85-87)

^§^n<=10 subjects

^*∗*^Hypertensive BP: SBP or DBP at or above the 95th age-, sex-, and height-specific percentile for children and adolescents under 18 years or BP ≥140/90 mmHg irrespective of the percentile; for adults aged 18 or older: ≥140/90 mmHg.

## Data Availability

The data used to support the findings of this study are available from the corresponding author upon request.
